# A Hybrid Differential Symbiotic Organisms Search Algorithm for UAV Path Planning

**DOI:** 10.3390/s21093037

**Published:** 2021-04-26

**Authors:** Lisu Huo, Jianghan Zhu, Zhimeng Li, Manhao Ma

**Affiliations:** College of Systems Engineering, National University of Defense Technology, Changsha 410073, China; huolisu09@nudt.edu.cn (L.H.); zhujianghan72@nudt.edu.cn (J.Z.); mhma@nudt.edu.cn (M.M.)

**Keywords:** unmanned aerial vehicle, path planning, differential evolution, symbiotic organism search, particle swarm optimization, evolutionary algorithm

## Abstract

Unmanned aerial vehicle (UAV) path planning is crucial in UAV mission fulfillment, with the aim of finding a satisfactory path within affordable time and moderate computation resources. The problem is challenging due to the complexity of the flight environment, especially in three-dimensional scenarios with obstacles. To solve the problem, a hybrid differential symbiotic organisms search (HDSOS) algorithm is proposed by combining the mutation strategy of differential evolution (DE) with the modified strategies of symbiotic organism search (SOS). The proposed algorithm preserves the local search capability of SOS, and at the same time has impressive global search ability. The concept of traction function is put forward and used to improve the efficiency. Moreover, a perturbation strategy is adopted to further enhance the robustness of the algorithm. Extensive simulation experiments and comparative study in two-dimensional and three-dimensional scenarios show the superiority of the proposed algorithm compared with particle swarm optimization (PSO), DE, and SOS algorithm.

## 1. Introduction

The unmanned aerial vehicles (UAV), as aircraft controlled by airborne computers or remote command centers, have been playing more and more important roles in modern society applications. With no need for pilots on the vehicle, UAVs can perform dangerous and complicated tasks under tough environments without being restrained by the physical and psychological conditions of pilots. Applications of UAVs can be seen in scenarios including flood relief, search and relief after earthquakes, geographic information collection, surveillance and reconnaissance, and many more scenarios to come. In recent years, studies on UAVs have greatly improved the effectiveness of UAVs in practical applications, especially in battlefield scenarios. UAV path planning, as one of the most important techniques in UAV autonomous formation and application in engineering, has also drawn much attention in the trend [1].

UAV path planning is primarily to find a feasible path for UAV under different environments with the aim of minimizing the cost and satisfying all the constraints. The problem can usually be defined as a large-scale optimization problem with many constraints [2], which could be challenging for traditional techniques to get the exact solution. Previous studies aiming at solving this problem may include graph-based methods [3], vision-based methods [4], mixed integer linear programming (MILP) methods [5], and evolution-algorithm-based methods [6]. Artificial neural network (ANN) methods sometimes are also applied in solving trajectory optimization problems for robots and UAVs [7,8]. Mini-batch gradient descent (MBGD) or stochastic gradient descent (SGD), for example, are two optimization algorithms usually used in machine learning. However, since the UAV path planning problems are often modeled as highly non-convex optimization problems with discontinuous domain of definition and multiple constraints, ANNs have usually been hybridized with other algorithms such as potential field method and heuristic algorithms [9].

Path planning has been proven to be the NP-hard problem, and the complexity increases rapidly as the size of the optimization problem grows [10]. To reduce the complexity, many kinds of metaheuristic algorithms have been applied to obtain satisfying solutions for UAV path planning problem [11,12,13,14]. Compared with non-heuristic methods, heuristic methods have strong ability to obtain high-quality solutions in path planning and are easier to implement [9]. Some well-known heuristic optimization algorithms often applied here include genetic algorithm (GA) [15], particle swarm optimization (PSO) [16], differential evolution (DE) [10], artificial bee colony (ABC) [17], ant colony optimization (ACO) [18], bat algorithm (BA) [19], and grey wolf optimizer (GWO) [20]. These algorithms have also been successfully applied to practical engineering optimization problems.

Evolutionary algorithms (EAs) have been demonstrated effective to solve path planning problems with an affordable time and moderate computation resources during recent years [21]. Among which DE and its variants have been widely used and modified to produce satisfying solutions for path planning problems. Compared with other evolutionary algorithms, DE has been frequently chosen to solve the large-scale optimization problems because it is straightforward and simple to implement, with just a few parameters and only taking a few lines to code the core part of the strategies while having an outstanding performance on unimodal, multimodal and other complex problems due to its powerful global search ability [22]. Zhang et al. proposed an improved differential evolution algorithm for 3D UAV online path planning, and compared the proposed algorithm with algorithms including classical DE, genetic algorithm (GA) and PSO, and demonstrated the competitiveness of the new algorithm [23]. A modified multi-population differential evolution algorithm (MMPDE) was proposed by Li et al. to solve the path planning of UAV, in which a multi-population framework and two new operators were adopted, and simulation experiments showed the good performance of this algorithm [24]. An improved constrained DE algorithm was proposed in the literature [10] for UAV global route planning, the algorithm combined the standard DE with a level comparison method, which enabled it to control the satisfactory level and deal with constraints. Similar algorithms derived from standard DE and used for UAV route planning can be seen in the literature [25,26].

Symbiotic organisms search (SOS) is a recently introduced swarm-intelligence-based algorithm inspired by symbiotic interaction between organisms in an ecosystem. The algorithm was initially developed to solve an optimization problem over a continuous search space [27]. Main processes of SOS include mutualism phase, which mimics mutualistic relationships where two organisms can benefit from each other through interactions, commensalism phase in which one organism benefits from another one through interactions while the other one receives nearly no benefit, and parasitism phase, in which one organism picks another one as a host and try to kill and assume its position after certain interactions. The SOS has been proven to be competitive in convergence speed and robustness in comparison with traditional metaheuristic algorithms such as GA, PSO, DE, and ABC [28]. Applications of SOS to UAV path planning problem can be seen in the literature [29], in which a modified symbiotic organism search algorithm based on the simplex method was proposed to solve the route planning problem and was demonstrated to be a strong and robust algorithm compared with other main swarm-intelligence algorithms.

The literature mentioned above provide some useful algorithms and inspirations for UAV path planning problems. However, it is still a long way from finding a perfect and universal path planning algorithm for different practical environments. Given the high dimension and complex constraints of the problem in most cases, especially under three-dimensional environment, where the cost functions and constraints could be too complex and require huge computational resources, hybridization of effective algorithms has become a trend [30]. The technique of hybridization is to combine multiple metaheuristic algorithms for taking advantage of the best features of algorithms while avoiding the shortcomings. The hybridization usually has better performance than their parent algorithms [31,32,33,34], which showed great potential of hybridization with different metaheuristic algorithms in practical optimization problems.

In this paper, a hybrid differential symbiotic organism search algorithm (HDSOS) is proposed based on the hybridization of DE and SOS. The UAV path planning environments are analyzed and modeled to formulate the cost function, where practical elements are taken into consideration and different parts of costs are included. Based on the analysis, the proposed algorithm is developed by combining one of the mutation strategies of DE with particular phases of SOS, and the concept of traction function is introduced. Moreover, a perturbation strategy is used to enhance the robustness of the algorithm. B-Spline curve is illustrated and used to smooth the original path derived from control points. Furthermore, simulation experiments are conducted among four algorithms including HDSOS, PSO, DE, and SOS under four two-dimensional and four three-dimensional scenarios, respectively, and the results and convergence curves are visualized for intuitive comparisons. Statistics from extensive repetitive experiments show the superiority of the proposed algorithm compared with other algorithms.

The remains of this paper are organized as follows: Section 2 gives a literature review of recent methods that have been applied in UAV path planning. Section 3 provides the environmental analysis and mathematical model of the path planning problem. Preliminary knowledge of DE and SOS are introduced and illustrated in Section 4. Then, the proposed algorithm HDSOS is detailed in Section 5. Subsequently, the application of B-Spline curves in smoothing the original path is given in Section 6. The simulation experiments and comparative analyses under different scenarios are conducted in Section 7. Section 8 concludes the paper.

## 2. Literature Review

Broadly implemented UAV path planning strategies can be classified into heuristic and non-heuristic methods. Non-heuristic methods mainly include dynamic programming, geometric algorithms, potential field, MILP, etc., whereas heuristic methods mainly include evolutionary algorithms, swarm-intelligence algorithms, and nature-inspired algorithms. With the tremendous increase in computing power, the latter kind of methods has seen a great period of development in recent three years.

As an example, a GWO-based algorithm was proposed by Radmanesh et al. in 2018 to find the optimal UAV trajectory in the presence of moving obstacles [35]. The assumption is that the UAV is equipped with the automatic dependent surveillance-broadcast and is provided with the position of intruder aircraft. The proposed approach used the formulation of dynamic Bayesian, distance-based value function, and GWO to solve the problem of path planning and collision avoidance for UAVs in the presence of fixed and moving obstacles in an uncertain environment. The experimental results obtained from several scenarios showed the effectiveness of the proposed approach. However, the approach was mainly analyzed and applied in two-dimensional scenarios. Another GWO-based approach was proposed by Qu et al. in [31], in which they proposed a novel hybrid algorithm called HSGWO-MSOS, which combined simplified GWO and modified SOS. Simulation experiments showed that the proposed algorithm can acquire feasible and effective routes successfully. Nevertheless, the design of the proposed algorithm lacked consideration of the intrinsic characteristics of the problem.

An improved PSO algorithm named GBPSO was proposed by Huang et al. to enhance the performance of three-dimensional path planning for UAV with fixed wings [36]. A competition strategy was introduced into the standard PSO to improve the convergence speed and the search ability of the particles. GBPSO was compared with some existing methods in two simulation scenarios and the results verified the effectiveness. Shao et al. also proposed an effective method based on PSO in the literature [37], where a chaos-based Logistic map was first adopted to improve the particle initial distribution. The constant acceleration coefficients and maximum velocity were designed to adjust to the optimization process and improve solution optimality. A Monte-Carlo simulation for UAV formation was conducted and the results proved the effectiveness of the method. However, the simulation environment was mainly terrain without many obstacles.

Another newly proposed method for UAV path planning is θ-MAFOA [38], which is an improved version of fruit fly optimization algorithm (FOA). The authors adopted a mutation adaptation mechanism to enhance the balance of FOA in terms of the exploitation and exploration ability. The proposed algorithm was used to find the optimal flyable path in three-dimensional terrain environments with ground defense weapons. B-spline curve was employed to obtain a smooth path. Liu et al. also proposed an evolution-algorithm-based approach for UAV path planning in terrain environments [39]. The algorithm was based on improved t-distribution and could effectively deal with the high computational complexity and low search efficiency problems. These approaches mainly focused on environments with unknown geographic information.

DE was adopted as the fundamental algorithm for UAV path planning in the literature [40]. In this algorithm, individuals were selected depending on their fitness values and constraint violations. The selected individuals were then used to make mutation, and the proposed algorithm searched around the best individual among the selected individuals. The designed mechanism improved the exploitation while maintained the exploration. Pan et al. proposed a hybrid differential evolution algorithm combining two modified variants of DE together called CIJADE [41]. Moreover, the parameters were updated according to a modified parameter adaptation strategy in each generation to improve the performance. However, these methods mainly focused on the improvement of algorithms without considering the practical characteristics of UAV path planning.

The literature above show that there are many techniques proposed so far for UAV path planning problems, many of them inspired by metaheuristic algorithms. To the best of our knowledge, however, there is no metaheuristic-based technique in the literature combining the strategy of DE with strategies of SOS in an innovative way for both two-dimensional and three-dimensional UAV path planning with multiple obstacles. Considering the excellent performance of SOS in exploitation stage and the strong ability of DE in exploring the solution space, we attempt to combine the advantages of these two algorithms together. Moreover, the practical characteristics of flyable path are also taken into consideration to improve the efficiency.

## 3. Problem Formulation

The problem to be solved in this paper is to quickly generate a flyable path for the UAV in a two-dimensional or three-dimensional environment full of fixed obstacles. The flyable path should not have any overlapped parts with obstacles, at the mean time the total distance should be as short as possible. Given the energy consuming nature of UAVs, there should not be too many unnecessary turns and swerves in the flyable path, i.e., the curvature cost should be as small as possible. To efficiently plan the paths for UAVs, the environment must be investigated and analyzed as comprehensive as possible. In this paper, we are about to deal with the environment as follows: in two-dimensional cases, the obstacles are simplified into polygons and circles, in some cases mixed; whereas in three-dimensional environments, the obstacles are simplified into prisms and cylinders, in some cases mixed. The problem modeling and the calculation of cost function are described in more details below.

### 3.1. General Model of UAV Path Planning

**Figure 1:**
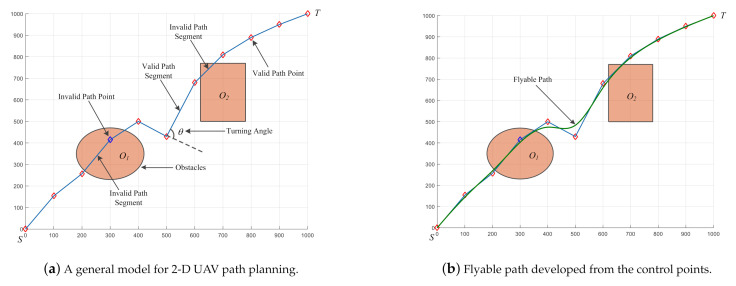
General model and flyable path for two-dimensional UAV path planning.

Path planning for UAVs consists of the following basic elements: the UAVs, the environments, the cost considerations, and the goals. Figure 1a shows a general model for a two-dimensional UAV path planning. Please note that the flying space is divided into a 2-D mesh.

Suppose that the UAV is about to fly from node *S*(0,0) to node *T*(1000,1000), $O_{1}$ and $O_{2}$ are obstacles in the mission space. Red and blue diamonds represent the path points which also known as control points. Blue lines connecting these points make up a possible solution for the UAV to fly. Since the scenario is constrained in a two-dimensional space, the blue diamond which falls into the obstacle is called invalid path point, correspondingly, the lines which overlap with the obstacles, i.e., $O_{1}$ and $O_{2}$, are called invalid path segments. All other red diamonds are valid path points and lines which do not overlap with obstacles are valid path segments. It is worth mentioning that in the real mission scenarios for UAVs, the flying paths are smooth curves other than polylines. Still, we usually need to confirm the path points which the UAV is about to pass through and connect them before we can smooth the whole path. θ is the turning angle for two adjacent path segments.

### 3.2. Cost Function and Analysis

Considering the actual flying scenario for UAVs, we assume that they need to travel from the starting point to the target point as fast as possible and at a minimal cost, which means no damage caused by the obstacles to the UAV bodies is preferable, and the total flight length should be as short as possible. Meanwhile, turning angle is supposed to be as small as possible, and there are yawing angle and pitch angle constraints, generally these angles are not supposed to be larger than certain constants [31].

It is obvious that the polyline ST shown in Figure 1a is not the actual flyable path for UAVs. There are many ways to obtain the flyable path from the control points, and the green curve $\tilde{ST}$ shown in Figure 1b provides an example of the flyable path derived from control points in Figure 1a.

The performance evaluation indicator of a flyable path is primarily composed of two parts: the threat cost $C_{threat}$ and the fuel cost $C_{fuel}$, while the fuel cost can be further divided into flying distance cost $C_{dist}$ and other operations cost, which is generally proportional to the curvature of the flyable path. Thus, we can represent the total cost of a possible path as follows:

(1) $$\begin{array}{cc} C_{all} & =C_{threat}+C_{fuel}\\ \; & =C_{threat}+C_{dist}+C_{curv}\\ \; & =\alpha \cdot \int_{0}^{length}J_{threat}dl+\beta \cdot \int_{0}^{length}J_{dist}dl+\gamma \cdot \int_{0}^{length}J_{curv}dl, \end{array}$$

where $C_{all}$ is the total cost of the flyable path, $C_{curv}$ is the curvature cost of the flyable path. $J_{threat}$, $J_{dist}$ and $J_{curv}$ represent the threat cost, distance cost and curvature cost on each segment of the flyable path, respectively, whereas α, β and γ are weighting parameters used to adjust the magnitude of each part of the cost.

Suppose that there are *N* segments $L_{1}$, $L_{2}$,⋯$L_{i}$…, $L_{N}$ in a polyline path before we turn it into a flyable path, and there are *M* obstacles $O_{1}$, $O_{2}$, …$O_{j}$…, $O_{M}$ in the environment. We use $L_{i}\cap O_{j}$ to denote the length of overlapped part between path segment $L_{i}$ and obstacle $O_{j}$, then the threat cost of each path segment $J_{threat,i}$ can be calculated as follows:

(2) $$J_{threat,i}=\sum_{j=1}^{M}L_{i}\cap O_{j}.$$

To calculate the distance cost of each flyable path segment, we can uniformly extract $N_{d}$ points on each segment $L_{i}$, denoted as $\left(x_{i,1},y_{i,1}\right),\left(x_{i,2},y_{i,2}\right),\ldots \left(x_{i,p},y_{i,p}\right)\ldots ,\left(x_{i,N_{d}},y_{i,N_{d}}\right)$, where $\left(x_{i,1},y_{i,1}\right)$ is the starting point of the segment and $\left(x_{i,N_{d}},y_{i,N_{d}}\right)$ is the ending point of the segment, then the distance cost of a flyable path segment can be calculated as in Equation (3). Please note that the three-dimensional distant cost can be calculated similarly, except that there are extra z-components.

(3) $$J_{dist,i}=\sum_{p=1}^{N_{d}-1}\sqrt{{\left(y_{p+1}-y_{p}\right)}^{2}+{\left(x_{p+1}-x_{p}\right)}^{2}}.$$

To calculate the curvature cost of a flyable path segment, we need to transform the original polyline path ST into a flyable path $\tilde{ST}$, and then uniformly extract $N_{i}$ points on each flyable path segment $L_{i}$, denoted as $\left(x_{i,1},y_{i,1}\right),\left(x_{i,2},y_{i,2}\right),\ldots \left(x_{i,k},y_{i,k}\right)\ldots ,\left(x_{i,N_{i}},y_{i,N_{i}}\right)$, then the curvature cost of a path segment can be represented as:

(4) $$J_{curv,i}=\sum_{k=1}^{N_{i}}\frac{|y_{i,k}^{\prime \prime }|}{{\left(1+y_{i,k}^{\prime 2}\right)}^{3/2}},$$

where $y_{i,k}^{\prime }$ is the first derivative of $y_{i,k}$ with respect to $x_{i,k}$ at the point $\left(x_{i,k},y_{i,k}\right)$ of the flyable path, whereas $y_{i,k}^{\prime \prime }$ is the second derivative of $y_{i,k}$ with respect to $x_{i,k}$ at the point $\left(x_{i,k},y_{i,k}\right)$ of the flyable path.

## 4. Preliminary Knowledge and Algorithms

### 4.1. Differential Evolution

DE [42] is an evolutionary algorithm proposed to solve global optimization problems over continuous spaces, and is arguably one of the best stochastic real-parameter optimization algorithms in current use [22]. By adding the difference of randomly chosen individuals in the population to current one, DE shows powerful performance in exploring the continuous solution space. DE and its major variants have been applied to multiobjective, large-scale, and constrained optimization problems. Main procedures of DE include initialization of the population, mutation with difference of individuals, crossover and selection. Among which the mutation stage is the essential operation. In this paper, one of the mutation strategies of DE variants, called target-to-best, is referenced and used in the design of the proposed algorithm. The strategy can be represented as follows:

(5) $$“DE/target-to-best/1^{”}:{\vec{V}}_{i,G}={\vec{X}}_{i,G}+F\cdot \left({\vec{X}}_{best,G}-{\vec{X}}_{i,G}\right)+F\cdot \left({\vec{X}}_{r_{1}^{i},G}-{\vec{X}}_{r_{2}^{i},G}\right),$$

where ${\vec{X}}_{i,G}$ is the *i*th individual in generation *G* and ${\vec{X}}_{best,G}$ is the best individual, indices $r_{1}^{i},r_{2}^{i}$ are mutually exclusive integers stochastically chosen from the current population range [1,NP]. *F* is the positive scaling factor used to scale the difference of individuals, and belongs to the open interval (0,1). Thus, we can obtain the new donor individual ${\vec{V}}_{i,G}$, which then exchanges its components with the target individual ${\vec{X}}_{i,G}$ to generate a trial individual. Based on the principle of greed, individuals with better fitness values will be selected to form a new generation.

### 4.2. Symbiotic Organisms Search

SOS is a recently designed robust and powerful metaheuristic algorithm for numerical optimization and engineering design problems [27]. Inspired by the biological interactions between organisms in an ecosystem, SOS elaborately imitates the natural process of mutualism, comensalism, and parasitism.

In the mutualism phase, the *i*th organism of the ecosystem $X_{i}$ is interacted with another randomly chosen organism $X_{j}$. New organisms generated from $X_{i}$ and $X_{j}$ can be calculated through following equations:

(6) $$X_{inew}=X_{i}+rand\left(0,1\right)*\left(X_{best}-Mutual\_Vector*BF_{1}\right),$$

(7) $$X_{jnew}=X_{j}+rand\left(0,1\right)*\left(X_{best}-Mutual\_Vector*BF_{2}\right),$$

(8) $$Mutual\_Vector=\frac{X_{i}+X_{j}}{2},$$

where $BF_{1}$ and $BF_{2}$ are benefit factors randomly determined as either 1 or 2, and $X_{best}$ is the best organism in the current ecosystem. The organisms are then updated if only the new ones fit better than the old ones.

The second phase is commensalism, in which organism $X_{i}$ attempts to benefit from another organism $X_{j}$ while $X_{j}$ neither benefits nor suffers from organism $X_{i}$. Organism $X_{i}$ is then updated only if its new fitness value surpasses its previous one. This process can be denoted as follows:

(9) $$X_{inew}=X_{i}+rand\left(-1,1\right)*\left(X_{best}-X_{j}\right).$$

In the parasitism phase of SOS, individual organism $X_{i}$ is duplicated, and then randomly modified in selected dimensions. The new generated organism is then compared with another randomly chosen organism in the ecosystem to see whether the chosen host can be replaced or killed, if the new one performs better, then the host will no longer exist in the ecosystem.

## 5. Proposed Hybrid Differential Symbiotic Organisms Search Algorithm (HDSOS)

DE is arguably one of the most competitive evolutionary algorithms with respect to global exploration ability. However, its performance at exploitation stage is usually flawed. In comparison with DE, SOS has excellent performance at exploitation stage, but relatively poor at the exploration stage for more possible solutions [2]. Therefore, in this paper, a new algorithm HDSOS is proposed by combining the mutation strategy of DE with several phases of SOS algorithm, thus to solve the two-dimensional and three-dimensional UAV path planning problems. The mutualism phase and commensalism phases of SOS are retained and modified for efficiency consideration while the parasitism phase is discarded. Taking the practical environment attributes into consideration, we define the traction function, which is used to pull the organisms toward a more promising direction in the evolutionary process. Traction function is combined with the *target-to-best* mutation strategy in DE to better explore the solution space. Furthermore, a perturbation strategy is used according to the fitness level of all the organisms in the ecosystem to provide more possibilities when the evolutionary process seems to stagnate.

### 5.1. Mainframe of HDSOS

To get the fittest individual in the population or ecosystem as fast as possible, the random search operation without a purpose during the evolutionary process should be reduced correspondingly. By analyzing and dividing the objective function, we can get a better understanding of the optimization objective, thus reduce the aimlessly search operations. Therefore, we can define the traction function $F_{t}$ here as any part of the objective function in Equation (1). Although the cost function has a linear relationship with its subitems, simulations show that the best individual usually at the same time has one of the best subitem values. Thus, we can define the traction function here as:

(10) $$F_{t}=\int_{0}^{length}J_{curv}dl=\sum_{i=1}^{N_{S}}\sum_{k=1}^{N_{i}}\frac{\left|y_{i,k}^{\prime \prime }\right|}{{\left(1+y_{i,k}^{\prime 2}\right)}^{3/2}},$$

where $N_{S}$ is the number of all path segments and $J_{curv}$ is the curvature cost of each path segment.

The mutualism phase in SOS is retained here in HDSOS because of its great potential in generating better new organisms, yet simplified for efficiency purpose. In this phase, individual $X_{i}$ and randomly chosen individual $X_{j}$ interact with each other to produce a mutual vector, then the mutual vector is used for the purpose of generating a better offspring $X_{ij\_new}$, which can be modeled in the following equations.

(11) $$X_{ij\_new}=X_{i}+rand\left(0,1\right)*\left(X_{best}-Mutual\_Vector*BF\right),$$

(12) $$Mutual\_Vector=\frac{X_{i}+X_{j}}{2},$$

where $X_{best}$ is the current global best individual with respect to objective function, and BF is the benefit factor, which is randomly determined as 1 or 2, imitating the natural phenomenon in which some organisms benefit better from the mutualism process than others. In contrast to the original SOS algorithm, which preserves both $X_{i}$ and $X_{j}$ to do comparison with the new organisms after interaction, HDSOS only preserves $X_{i}$, and the newly generated organism is regarded as an offspring of both $X_{i}$ and $X_{j}$. Thus, the calculation and comparison operations are largely reduced in this phase. The new offspring $X_{ij\_new}$ is then compared with $X_{i}$ by calculating the objective function, and $X_{i}$ will be replaced only if fitness value of the offspring outperforms $X_{i}$.

The second phase of HDSOS is commensalism phase, which is to benefit $X_{i}$ with the randomly chosen individual organism $X_{j}$. In the HDSOS, the commensalism phase is modified to better improve the fitness of $X_{i}$. The traction function value of each organism is evaluated, and the corresponding best organisms are chosen to pull $X_{i}$ to a promisingly better direction. In addition, the mutation strategy of DE is combined with the commensalism operation to explore the solution space more thoroughly. This operation can be denoted by the following equation.

(13) $$X_{inew}=X_{i}+rand\left(0,1\right)*\left(X_{Ft\_best,k}-X_{i}\right)+F*\left(X_{i}-X_{j}\right),\forall k\in \left\{1,2,\ldots ,N_{b}\right\},$$

where $X_{Ft\_best,k}$ is the *k*th best organism with respect to traction function $F_{t}$, and is chosen randomly from the first $N_{b}$ best organisms here. *F* is the positive control parameter for scaling the difference of $X_{i}$ and $X_{j}$ in the mutation operation and usually belongs to (0,1). Similarly, the obtained new organism will then be compared with $X_{i}$ with respect to the objective function, and $X_{i}$ will be replaced only when the fitness value of $X_{inew}$ surpasses that of $X_{i}$.

Another strategy used in HDSOS is the perturbation. This operation will only be applied when the evolutionary process seems to stagnate for certain generations. In this case, all organisms in the ecosystem will be evaluated and sorted according to their objective function values. Afterwards, a certain number of the worst organisms will be permanently vanished from the ecosystem, at the same time, equal number of organisms will be randomly generated and added into the ecosystem as part of the new generation. Suppose that after certain generations of stagnation, the perturbation operation is triggered. Assume that the upper and lower bounds for each organism are $U_{i}$ and $L_{i}$, respectively, then for each organism $X_{iw}$ from the $N_{w}$ worst organisms in the ecosystem, it will be replaced using the following formula:

(14) $$X_{iw}=rand\left(0,1\right)*\left(U_{i}-L_{i}\right)+L_{i}.$$

**Figure 2:**
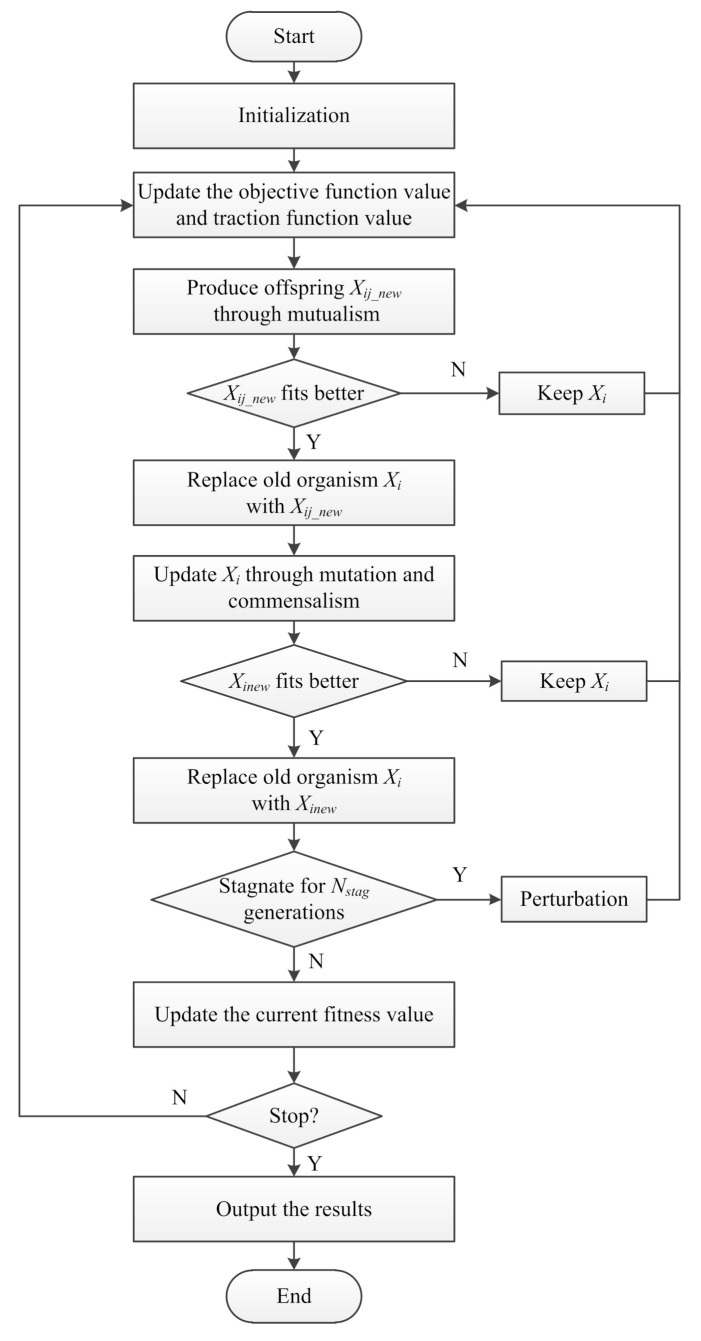
Flowchart of HDSOS.

Based on the aforementioned procedures and analyses, the mainframe of HDSOS can be further described in Algorithm 1 and Figure 2.
**Algorithm 1:** The main procedure of Hybrid Differential Symbiotic Organisms Search (HDSOS)
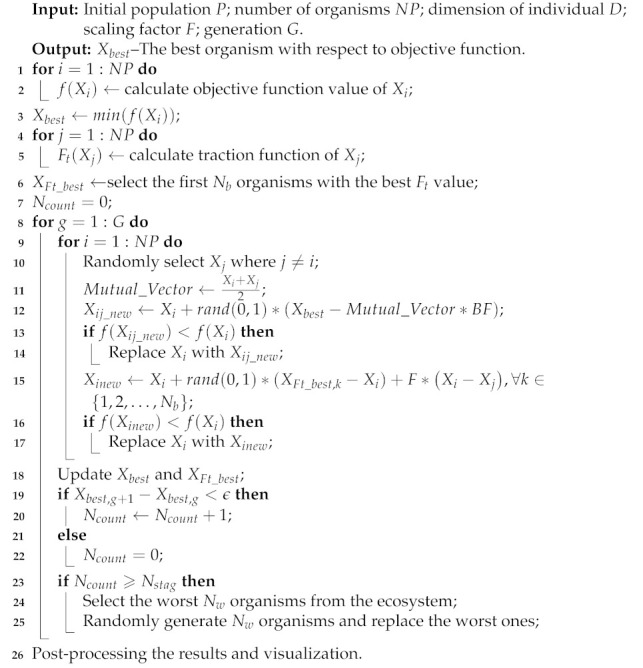


### 5.2. Application of HDSOS in Practical UAV Path Planning

**Figure 3:**
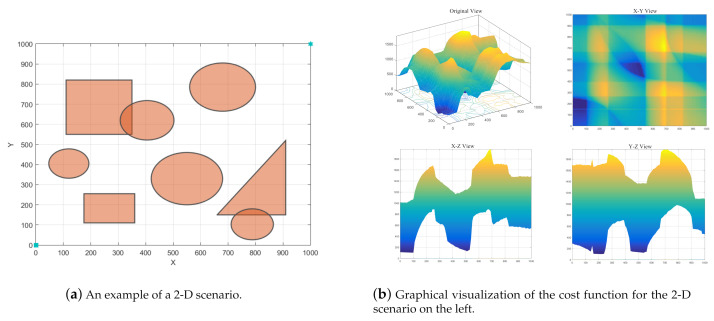
Example of a 2-D scenario and visualization of its cost function ($Dim(X_{i})=2$).

According to the analyses in Section 3.2, the cost function for UAV path planning could be complicated, especially when many control points are needed to determine a flyable path, which turns the case into a high-dimensional problem. For example, in a two-dimensional path planning scenario as shown in Figure 3a, the cost function could be intractable even when the decision variable is a two-dimension vector. The corresponding graphical view of the cost function for this scenario is displayed in Figure 3b. The figure shows that the objective function has irregular functional image, and there are several local minimum points, which could be misleading for general evolutionary algorithms. Moreover, the X−Z view and Y−Z view show that even for the local minimum points, they could be in a basin area which is relatively flat, and this will further impede the process for EAs to find the global minimum rapidly.

In general, two-dimensional or three-dimensional UAV path planning is to minimize the cost function as shown in Equation (1). Through proper preprocessing, the equation can be applied to both two-dimensional and three-dimensional scenarios. For two-dimensional scenarios, threat cost $C_{threat}$ can be calculated from all the path segments that overlap with obstacles, whereas in three-dimensional scenarios, threat cost can also be calculated from all the path segments that pass through three-dimensional obstacles. The ways to obtain distance cost $C_{dist}$ are similar in both kinds of scenarios. For the curvature cost $C_{curv}$, Equation (4) is practicable to three-dimensional scenarios if we properly represent the feasible solutions. In this paper, once the start and target points are determined, one way to represent a possible solution in three-dimensional scenarios is to uniformly divide the space into a set of cubic grids, then decide the coordinates on two axes in advance according to the dimensions of the decision variable. Please note that by this way, the torsion cost of the path curve could be transformed into the curvature cost, which is proved to be an efficient way in solving path planning problem here.

However, to get a flyable path as illustrated in Figure 1, we need to process the output results of the HDSOS algorithm to satisfy the physical properties of UAVs. Specific method adopted to smooth the original path will be detailed in the following sections.

## 6. Path Smoothing Method

Generally, the original paths obtained by evolutionary algorithms are not suitable for practical UAV flight, since they are usually continuous polylines but non-differentiable. To ensure the path is flyable and smooth, special techniques are needed here. B-Spline curve smoothing strategy is introduced and used to dynamically smooth the paths generated by HDSOS. The B-Spline curves have evolved from Bezier curves, and are highly appropriate in the real-time path planning for UAVs since they need only a few variables to define complicated curves [2]. The advantages of Bezier curves, such as geometrical invariability, convexity-preserving, and affine invariance, are all inherited by B-Spline curves [31,43].

B-Spline curves are constructed based on base functions. Suppose that there are n+1 control points for the curve with coordinates $\left(x_{0},y_{0},z_{0}\right),\ldots ,\left(x_{n},y_{n},z_{n}\right)$, then the coordinates of the B-Spline curve can be denoted as:

(15) $$\left\{\begin{array}{c} x\left(u\right)=\sum_{i=0}^{n}x_{i}\cdot N_{i,k}\left(u\right)\\ y\left(u\right)=\sum_{i=0}^{n}y_{i}\cdot N_{i,k}\left(u\right)\\ z\left(u\right)=\sum_{i=0}^{n}z_{i}\cdot N_{i,k}\left(u\right) \end{array}\right.,$$

where *u* is the free parameter of the curve, $\left(x_{i},y_{i},z_{i}\right)$ are the control points, and $N_{i,k}\left(u\right)$ are the k-order normalized B-Spline base functions defined by the following Cox-de Boor recursion:

(16) $$\left\{\begin{array}{c} N_{i,0}=\left\{\begin{array}{c} 1,\;\;if\;\;u_{i}⩽u⩽u_{i+1}\\ 0,\;\;otherwise \end{array}\right.\\ N_{i,k}\left(u\right)=\frac{u-u_{i}}{u_{i+k}-u_{i}}N_{i,k-1}\left(u\right)+\frac{u_{i+k+1}-u}{u_{i+k+1}-u_{i+1}}N_{i+1,k-1}\left(u\right) \end{array}\right.,$$

where $U=u_{0},\ldots ,u_{m}$ is called knot vector, of which the most common form is the uniform non-periodic one, denoted as follows:

(17) $$u_{i}=\left\{\begin{array}{c} 0,\;\;\;\;if\;i<k+1\\ i-k,\;\;if\;k+1⩽i⩽n\\ n-k+1,\;if\;n<i \end{array}\right..$$

**Figure 4:**
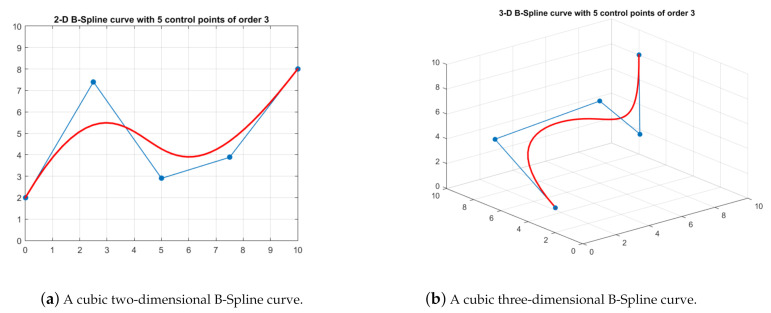
The cubic B-Spline curves and their control polygons.

Figure 4 shows the B-Spline curves in two-dimensional and three-dimensional spaces, all with five control points of order 3. Compared with the Bezier curves, B-Spline curves overcome the disadvantage that changing a control point will affect the entire curve. Furthermore, the degree of the polynomial would not increase no matter how many control points are used.

## 7. Simulation Experiments and Results

In this section, the proposed algorithm HDSOS is evaluated under eight different scenarios, i.e., four two-dimensional scenarios and four three-dimensional scenarios. To show the superiority of HDSOS, three popular metaheuristic algorithms are adopted here for comparison, including PSO, DE, and SOS. The latter two algorithms are related to the proposed algorithm. For the fair comparison in terms of run times and performance, all the experiments are conducted in MATLAB R2015a on Windows 10 operating system, with Core i7-6500U 2.60 GHz CPU, 8 GB memory.

For the two-dimensional scenarios, obstacles are placed in a field with the size of 1000 × 1000 square units. The starting point is set to (0,0) and the target point (1000,1000). Scenario A of the two-dimensional field has obstacles denoted by filled polygons, scenario B by filled circles, whereas scenario C and scenario D have both kinds of obstacles mixed, and scenario D has more densely distributed obstacles. For the three-dimensional scenarios, obstacles are represented by prisms and cylinders and are placed in a space with the size of 1000 × 1000 × 1000 cubic units. The starting point is set to (0,0,0) and the target point (1000,1000,1000). Scenario E is a three-dimensional space with obstacles represented by prisms, scenario F by cylinders, whereas scenario G and scenario H have both kinds of obstacles scattered in the space, and scenario H has more densely distributed obstacles.

It is worth mentioning that while the polyline corresponding to the control points is guaranteed to avoid the obstacles with the optimization procedure, the B-spline curve that results from the path smoothing method might not avoid the obstacles. There are basically two solutions to address this issue. The first solution is to increase the dimension of the decision variable, i.e., number of control points, to ensure that the smoothed path is as close to the original polyline path as possible. However, this may result in a prominent increase in computational complexity and time consumption. Another efficient solution is to set a buffer zone around each obstacle, which would effectively reduce the possibility that the smoothed path might not avoid obstacles. In this article, the latter solution is adopted in the optimization process to achieve this purpose. In both two-dimensional and three-dimensional scenarios, obstacles have buffer zones around them with the width of 5, thus to further improve the performance of the algorithm.

The dimension of the decision variable $X_{i}$ is set to 10 in all scenarios. All three existing algorithms and the proposed algorithm HDSOS will be conducted for 30 independent runs under each of the eight scenarios. Since all the four algorithms are population-based evolution, the number of the population is set to 50 for all, furthermore, the generation is set to 50 for all. The comparative results under each scenario are analyzed and compared in terms of mean value, standard deviation and runtime. Moreover, for each scenario, representative optimization results will be visualized and the convergence curves of four algorithms will be shown in figures for intuitive comparisons.

### 7.1. Experiments and Comparisons under Two-Dimensional Scenarios

**Figure 5:**
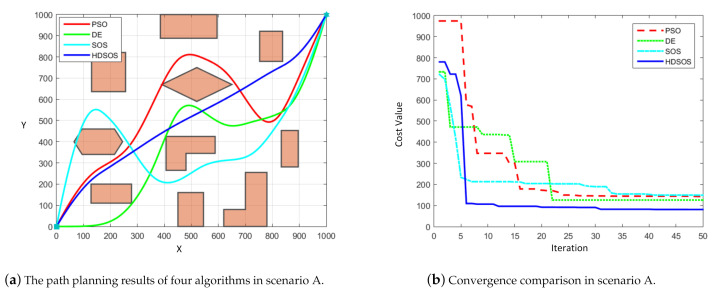
The comparative results for two-dimensional scenario A.

For scenario A with polygon obstacles, Figure 5 shows one of the optimization results of four algorithms. We can see from Figure 5a that the flyable paths obtained by PSO, DE and HDSOS can satisfy the requirements without crossing any obstacles while the paths planned by SOS is a failure. Among the three successful paths, the one planned by HDSOS is obviously optimal compared with the other two paths, with less swerves and shorter length. The convergence curves of the four algorithms are shown in Figure 5b. Through the iteration, HDSOS rapidly converges to near-optimal level in less than 10 iterations, and stays at the optimal value of 81.16 after iteration 31. DE has the suboptimal performance in this case with the final cost value 125.97 after iteration 22. PSO also converges rapidly in the first 20 iterations, but stays at a relatively high value of 142.73 during the last half of iterations. SOS, however, performs worst in this case with an unflyable path and a high cost value of 149.24 at the end of the iteration.

**Figure 6:**
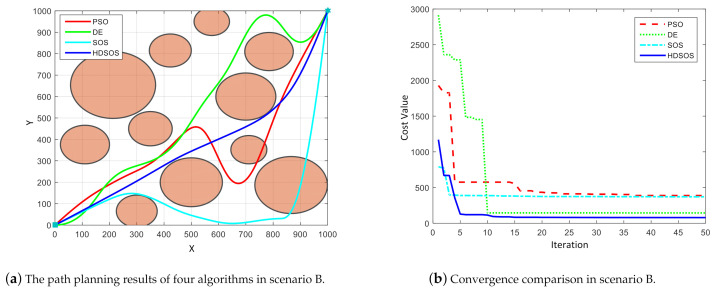
The comparative results for two-dimensional scenario B.

For scenario B with circle obstacles, Figure 6 shows one of the optimization results of final paths and corresponding convergence curves. In this case, only the paths generated by HDSOS and DE are flyable whereas the other two are failures, of which the final trajectories pass through the obstacles, as shown in Figure 6a. Moreover, the trajectory obtained by HDSOS is smoother with shorter total length than that by DE. We can see from Figure 6b that the HDSOS again has the rapidest rate of convergence, in less than 10 iterations, it converges to the near-optimal value and stays at 79.39 at the early stage. Compared with HDSOS, SOS algorithm has a relatively small value at the initial stage, but is trapped in the local minimum after several iterations and finally stays at a high value of 370.75. Compared with PSO, DE converges faster but is also trapped in local minimum and has the final value of 144.89. PSO, however, has the worst performance in this case with a high convergence value of 387.81.

**Figure 7:**
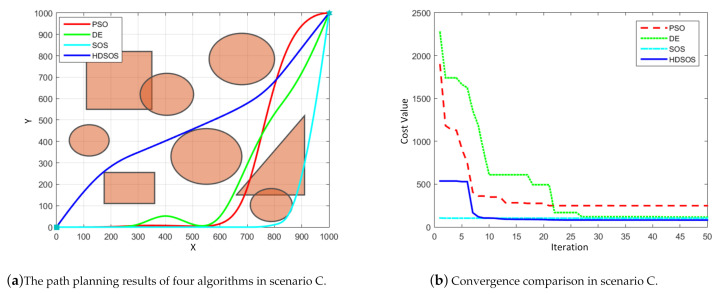
The comparative results for two-dimensional scenario C.

Scenario C has mixed obstacles of prisms and cylinders, Figure 7 shows one of the optimization results. In this case, we can see from Figure 7a that HDSOS and DE are the two algorithms that finally obtain the flyable paths while the other two fail. Compared with the path generated by DE, the path obtained by HDSOS is smoother and with shorter total length. Figure 7b shows that HDSOS converges in less than 10 iterations with the smallest final value of 80.66. Algorithm SOS, although with the smallest value at initial stage, is trapped in the local minimum and finally surpassed by HDSOS, with the final convergence value of 102.53 and an unflyable trajectory. PSO again has the worst performance in this case with the final convergence value of 248.19.

**Figure 8:**
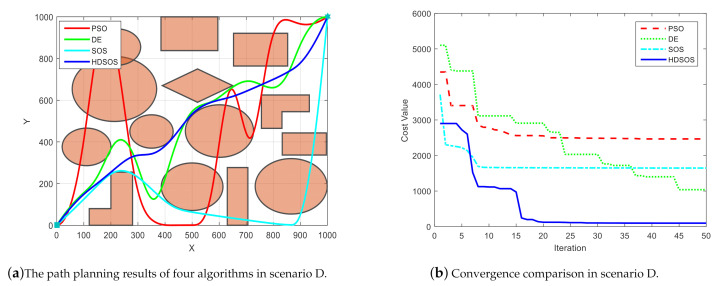
The comparative results for two-dimensional scenario D.

Scenario D has dense and mixed obstacles, which is rather difficult for general methods to find a flyable path. Figure 8 shows one of the final comparative results of the four algorithms. In this case, however, HDSOS is the only algorithm that finds a flyable path while others all fail, as shown in Figure 8a. The path generated by SOS crosses the most obstacles, and the path generated by DE crosses the least obstacles. Figure 8b shows that HDSOS converges quickly to its optimal value in iteration 20, and stays at a very small cost value of 96.12 afterwards. SOS converges pretty fast at the early stage, but is trapped in some local optimal point within 10 iterations, and finally stays at a high cost value of 1646.59. DE performs better compared with SOS in this case, it converges continuously throughout the iteration process, but with a slow speed and a final cost value of 1038.22. PSO is also trapped in some local optimal point at very early stage, and stays at a high cost value of 2466.02 after iteration 21, the corresponding path has several unnecessary big swerves and a long total distance as well.

**Figure 9:**
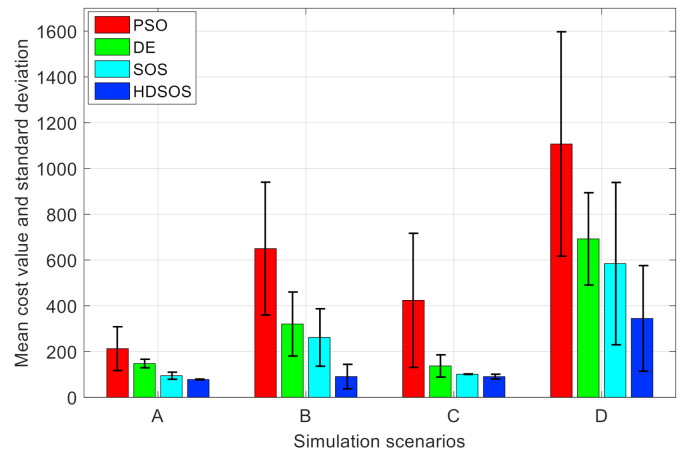
Performance comparison of algorithms among scenarios.

Table 1 shows the statistical results of the four algorithms in 30 independent runs under each scenario, and the results are further illustrated in Figure 9. It can be seen that HDSOS outperforms any other algorithms in all the four two-dimensional scenarios. In scenario A, HDSOS has the best mean cost value and a very small standard deviation of only 1.72. SOS has the second-best mean value and standard deviation, but the runtime is nearly twice as long as that of HDSOS. PSO, however, has the worst performance with respect to mean value and standard deviation, although it has the shortest runtime. In scenario B, HDSOS is significantly superior compared with other algorithms in terms of mean value and stability. DE, of which the runtime is as long as that of HDSOS, obtains much larger mean cost value and standard deviation. In scenario C, HDSOS again has the best mean value compared with other algorithms, although the standard deviation is a bit larger than that of SOS. PSO still has the worst mean value and standard deviation. In scenario D, which is the most challenging one among all four two-dimensional scenarios, HDSOS still has the best performance with respect to the mean cost value, although the standard deviation of DE is smaller than that of HDSOS, the mean cost value of DE is more than twice as that of HDSOS, while the two algorithms have nearly the same runtime. SOS has the second-best performance with respect to the mean cost value; however, it runs almost twice as long as algorithm HDSOS or DE. Algorithm PSO has the least runtime, but the mean cost value and standard deviation are more than twice as large as those of HDSOS.

### 7.2. Experiments and Comparisons under Three-Dimensional Scenarios

**Figure 10:**
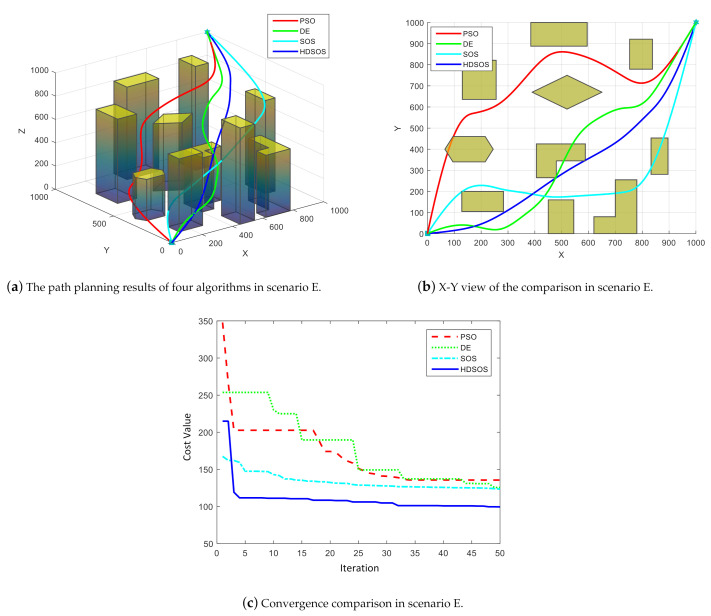
The comparative results for three-dimensional scenario E.

Three-dimensional scenario E contains many prismatic obstacles. Figure 10 shows one of the final path planning results of the four algorithms, where Figure 10a is the three-dimensional overview of the results, Figure 10b is the X-Y view of the final results and Figure 10c shows the convergence curves of the four algorithms. We can see from the upper two figures that in this case, HDSOS, SOS and DE all get the flyable path, whereas the path generated by PSO is a failure. Among the three flyable paths, the one acquired by HDSOS has the least swerves and the shortest total length, i.e., the smallest cost value. The comparison of the convergence curves clearly testifies the superiority of HDSOS over other algorithms. The HDSOS converges rapidly to its near-optimal value in less than 5 iterations and finally gets a satisfying value of 99.40. PSO and DE, on the other hand, converge rather slow and obtain the final values of 135.54 and 125.54, respectively. SOS also converges at a slow rate, although it has the smallest cost value after the first iteration, but stays at a relatively high value of 123.40 at the final stage.

**Figure 11:**
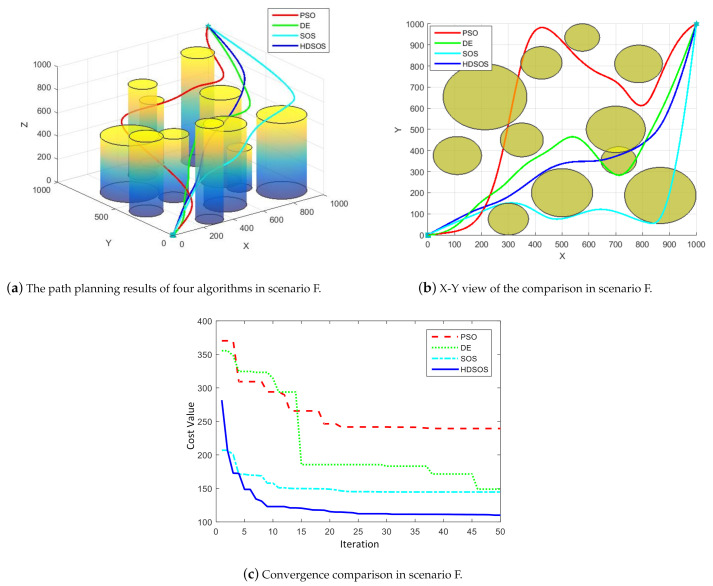
The comparative results for three-dimensional scenario F.

For three-dimensional scenario F, which contains many cylinder obstacles, Figure 11 shows one of the final path planning results of the four algorithms. We can see from Figure 11a,b that the paths attained by HDSOS, DE and SOS are all flyable, with the path acquired by HDSOS being the shortest and optimal one. The path attained by PSO, however, passes through more than one-cylinder obstacles, which means it is a failure. The convergence curves show the details of the four algorithms through iterations. The HDSOS converges quickly at the early stage and finally stays at its optimal value of around 110.09. Algorithm SOS acquires a relatively small value at the first iteration but converges slowly and end up with the cost value of 144.64. DE also converges at a low rate and remains a high value of 148.93 at the end of the iteration. PSO once again has the lowest rate of convergence and acquires the worst results at the end of the iteration.

**Figure 12:**
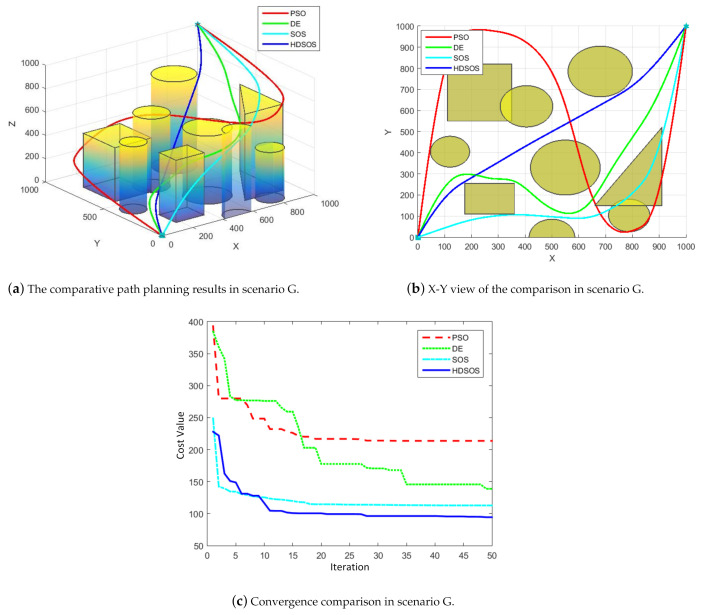
The comparative results for three-dimensional scenario G.

For three-dimensional scenario G with both prismatic and cylinder obstacles, Figure 12 shows one of the final results acquired by the four algorithms. In this case, we can see from the upper two figures that all the four paths obtained by these algorithms are flyable without passing through any obstacle. However, the HDSOS still outperforms the other algorithms in terms of distance cost and curvature cost. The convergence curves prove that HDSOS converges quickly to its near-optimal value and stays at 94.36. SOS has the second-best final cost value of 112.76. Although DE gets the final value of 138.79 with a slow speed, PSO again obtains the worst cost value of 213.67, which we can see from the final path with several big swerves that are indeed unnecessary.

**Figure 13:**
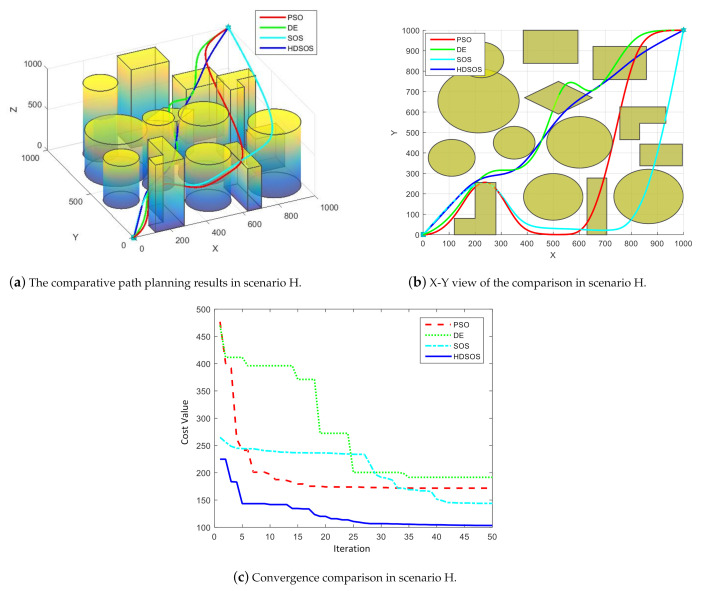
The comparative results for three-dimensional scenario H.

For three-dimensional scenario H with dense obstacles including prisms and cylinders, Figure 13 shows one of the final path planning results of the four algorithms. We can see from Figure 13a,b that in this case, only algorithm HDSOS finally gets the flyable path whereas all other algorithms fail. The path acquired by HDSOS is direct and efficient, and with the shortest total length. The paths acquired by other algorithms all cross some obstacles at several certain path segments. The convergence curves as shown in Figure 13c further testify the competitiveness of the proposed algorithm. HDSOS quickly finds solution with a relatively small cost value at the early stage, and continues to converge to the global optimal point afterwards, and finally gets a very small cost value of 103.38. The DE and SOS have poor convergence rates and finally stay at high cost values of 191.67 and 143.90, respectively. PSO has pretty fast convergence rate at the early stage, but is soon trapped in some local minimum within 20 iterations, and acquires the second worst cost value of 171.77 at the end of the iteration.

**Figure 14:**
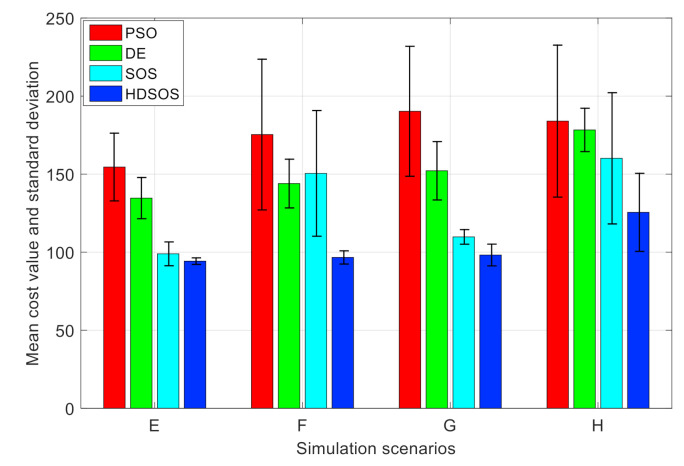
Performance comparison of algorithms among scenarios.

Table 2 and Figure 14 show the statistical results of the four algorithms in each three-dimensional scenario. The histogram shows clearly that the PSO performs worst whereas HDSOS performs best in all the four scenarios. In scenario E, HDSOS gets the smallest mean value and standard deviation of 94.27 and 2.11, respectively. DE, while requires as much time as HDSOS, obtains much larger mean value and standard deviation. Algorithm SOS has the second-best performance but it requires nearly twice as long as HDSOS. PSO has the worst performance, although it requires less time than other algorithms. In scenario F, HDSOS obtains the smallest mean value of 96.65 and standard deviation of 4.24, which are much smaller than any other algorithms here. However, in this scenario, DE outperforms SOS in terms of all the three indices. In scenario G, with HDSOS being the best at mean value, SOS has a slight advantage over HDSOS in terms of standard deviation. PSO remains the worst and DE remains the second worst in terms of mean value and standard deviation. In scenario H, HDSOS has the smallest mean cost value and the second-best standard deviation value, and only takes half as much time as that of SOS. Although DE has the smallest standard deviation value, it has much larger mean value than HDSOS. PSO again has the worst performance among all the four algorithms. Table 2 and Figure 14 once again testify the superiority of HDSOS over other algorithms.

### 7.3. Advantages and Limitations of HDSOS

Experimental studies above show that the proposed HDSOS has strong capability in generating flyable paths in both two-dimensional and three-dimensional scenarios. The combination of the traction function and the mutation strategy of DE gives the proposed algorithm excellent ability in exploring the solution space at the early stage, whereas the adopted mutualism and commensalism phases of SOS enable the algorithm to avoid being trapped in local optimality at the later stage. The proposed algorithm has very stable performance in terms of mean value and standard deviation, and can always get the flyable path in limited iterations compared with other algorithms. Moreover, the time consumption of HDSOS is satisfying due to the simplicity of the strategy.

However, there are still limitations for the proposed algorithm. First, the implementation of HDSOS in UAV path planning needs an extra traction function, which should be defined in advance, making the algorithm not very convenient in general use. Second, the calculation of traction function also adds extra execution time, reducing efficiency of the algorithm to some extent. Third, the algorithm is mainly designed for environments with solid obstacles, and usually with the same dimension of the environment, i.e., two-dimensional obstacles for two-dimensional environments, and three-dimensional obstacles for three-dimensional environments. Therefore, the algorithm might not be efficient for two-dimensional environments with linear obstacles or three-dimensional environments with planar obstacles.

## 8. Conclusions and Future Work

In this paper, a new algorithm called hybrid differential symbiotic organisms search (HDSOS) is proposed to solve the UAV path planning problem under two-dimensional and three-dimensional scenarios. The mutualism phase and parasitism phase of SOS are modified and adopted in the new algorithm in consideration of the local search capability of SOS, the concept of traction function is introduced to improve the efficiency of the algorithm. To enhance the global search ability of the algorithm, mutation strategy of DE is adopted and combined with the algorithm in commensalism phase. Furthermore, a perturbation strategy is applied when the evolutionary process seems to stagnate for certain generations, thus to improve the robustness of the algorithm. B-Spline curves technique is illustrated and used to smooth the initial path acquired by the algorithm. To demonstrate the superiority of the proposed algorithm, comparative experiments are conducted among HDSOS and another three algorithms including PSO, DE and SOS. Simulation results and analysis under four two-dimensional scenarios and four three-dimensional scenarios show that the proposed algorithm is pretty competitive and efficient compared with other algorithms aforementioned.

In our future work, we will be interested in studying the physical attributes of UAVs and the influence they may have on UAV path planning. Moreover, multi-UAV path planning and joint mission will also be of the interests of our research.

## Figures and Tables

**Table 1:** Performance of algorithms in two-dimensional scenarios (30 runs).

Algorithms	Scenario A			Scenario B			Scenario C			Scenario D		
	Mean	Std	Runtime(s)	Mean	Std	Runtime(s)	Mean	Std	Runtime(s)	Mean	Std	Runtime(s)
PSO	212.80	95.72	32.80	649.72	290.22	17.36	423.77	292.97	18.33	1106.98	490.29	31.5473
DE	147.57	18.76	64.96	320.29	139.89	34.39	137.07	48.67	38.91	692.31	201.68	62.4913
SOS	94.49	15.58	129.52	261.47	125.53	67.98	101.24	1.17	55.61	584.27	354.58	123.903
HDSOS	77.92	1.72	64.46	90.67	53.40	34.09	90.48	10.40	34.86	344.75	230.92	63.5827

**Table 2:** Performance of algorithms in three-dimensional scenarios (30 runs).

Algorithms	Scenario E			Scenario F			Scenario G			Scenario H		
	Mean	Std	Runtime(s)	Mean	Std	Runtime(s)	Mean	Std	Runtime(s)	Mean	Std	Runtime(s)
PSO	154.56	21.69	28.04	175.34	48.27	15.00	190.27	41.62	18.19	183.96	48.67	25.043
DE	134.64	13.20	55.25	143.96	15.60	29.84	152.15	18.70	36.53	178.33	13.89	50.738
SOS	98.96	7.62	107.98	150.47	40.26	57.43	109.79	4.69	69.42	160.13	42.02	97.9167
HDSOS	94.27	2.11	53.84	96.65	4.24	30.62	98.21	6.97	35.87	125.52	24.99	51.3263

## Data Availability

All data generated or analyzed during this study are included in this published article.

## Abbreviations

ABC	Artificial Bee Colony
ACO	Ant Colony Optimization
ANN	Artificial Neural Network
BA	Bat Algorithm
DE	Differential Evolution
EA	Evolutionary Algorithm
FOA	Fruit Fly Optimization Algorithm
GA	Genetic Algorithm
GWO	Grey Wolf Optimizer
HDSOS	Hybrid Differential Symbiotic Organisms Search
HSGWO-MSOS	Hybrid Simplified Grey Wolf Optimizer and Modified Symbiotic Organisms Search
MBGD	Mini-batch Gradient Descent
MILP	Mixed Integer Linear Programming
MMPDE	Modified Multi-population Differential Evolution Algorithm
PSO	Particle Swarm Optimization
SGD	Stochastic Gradient Descent
SOS	Symbiotic Organisms Search
UAV	Unmanned Aerial Vehicle
